# Acid base variables predict survival early in the course of treatment with continuous venovenous hemodiafiltration

**DOI:** 10.1097/MD.0000000000012221

**Published:** 2018-09-07

**Authors:** Rogério da Hora Passos, Juliana Ribeiro Caldas, João Gabriel Rosa Ramos, Paulo Benigno Pena Batista, Danilo Teixeira Noritomi, Nelson Akamine, Marcelino de Souza Durão Junior, Bento Fortunato Cardoso dos Santos, Virgilio Gonçalves Pereira Junior, Julio Cesar Martins Monte, Marcelo Costa Batista, Oscar Fernando Pavão dos Santos

**Affiliations:** a Hospital São Rafael—Salvador Ba; b Departamento de Doentes Graves—Hospital Israelita Albert Einstein—São Paulo; c Hospital Português—Salvador-Bahia—Brazil.

**Keywords:** acidosis, acute kidney injury, hemodiafiltration, quantitative analysis

## Abstract

Metabolic acid–base disorders, especially metabolic acidosis, are common in critically ill patients who require renal replacement therapy. Continuous veno-venous hemodiafiltration (CVVHDF) achieves profound changes in acid–base status, but metabolic acidosis can remain unchanged or even deteriorate in some patients. The objective of this study is to understand the changes of acid–base variables in critically ill patients with septic associated acute kidney injury (SA-AKI) during CVVHDF and to determine how they relate to clinical outcome.

Observational study of 200 subjects with SA-AKI treated with CVVHDF for at least 72 hours. Arterial blood gases and electrolytes and other relevant acid–base variables were analyzed using quantitative acid–base chemistry.

Survivors and nonsurvivors had similar demographic characteristics and acid–base variables on day one of CVVHDF. However, during the next 48 hours, the resolution of acidosis was significantly different between the 2 groups, with an area under the ROC curve for standard base excess (SBE) and mortality of 0.62 (0.54–0.70), this was better than APACHE II score prediction power. Quantitative physicochemical analysis revealed that the majority of the change in SBE was due to changes in Cl and Na concentrations.

Survivors of SA-AKI treated with CVVHDF recover hyperchloremic metabolic acidosis more rapidly than nonsurvivors. Further study is needed to determine if survival can be improved by measures to correct acidosis more rapidly.

## 1 Introduction

Acid–base disorders especially metabolic acidosis are common in patients with acute kidney injury (AKI) requiring renal replacement therapy.^[1–3]^ While continuous venovenous hemodiafiltration (CVVHDF) is effective in correcting acid–base imbalance, some patients resolve metabolic acidosis more slowly than others and some are refractory to treatment.^[4–6]^ Emerging evidence suggests that metabolic acidosis can produce a variety of effects across a wide range of cellular process and is associated with prolonged hospital, intensive care length stay and poor prognosis. Some forms of metabolic acidosis appear to be stronger predictors of mortality than others.^[7–9]^ Thus, we sought to determine whether the type of metabolic acidosis and/or the rate of resolution predict hospital mortality in a cohort of critically ill patients with sepsis associated acute kidney injury, treated with CVVHDF.

## 2 Methods

### 2.1 Patients and data collection

This study was approved by the Investigational Review Board of the Hospital Israelita Albert Einstein. This retrospective study included patients admitted to a 45-bed medical-surgical department of intensive care during a 4.5-year period (January 1, 2002–July, 2006) who developed SA-AKI requiring renal replacement therapy for at least 72 hours. The indications for renal replacement therapy included refractory acidosis, refractory hyperkalemia, hemorrhage due to azotemia, uremic encephalopathy, uremic pericardial effusion, and hypervolemia. The data needed for analysis of the ICU patients were originally collected by the ICU staff as part of standard patient care, and are electronically stored and available for computer-based retrieval. We thus obtained demographic data (age, sex, APACHE II score, ICU mortality, 28 days mortality, and admission diagnosis) and biochemical data from our electronic ICU database. The biochemical data used were from the first and third day of CVVHDF.

### 2.2 Renal replacement therapy and anticoagulation

Vascular access was obtained via a 12-F double-lumen catheter (Arrow-Howes multiple-lumen hemodialysis catheter, Arrow International, Reading, PA) introduced into the jugular, subclavian, or femoral vein. Hemodiafiltration was performed using a M100 dialyser (Gambro, Lakewood, CO) on a Prisma machine with blood flow rate (*Q*_B_) = 100 mL/min; dialysate flow rate (*Q*_D_) = 2500 mL/h; and fluid reposition = 500 mL/h. The replacement fluid for hemofiltration consisted of 120 mmol/L Na, 120 mmol/L Cl, and 0.5 mmol/L Mg. The composition of the dialysate used during HDF was bicarbonate individualized 36 to 40 mmol/L, 140 mmol/L sodium, 2 mmol/L potassium, and 108 mmol/L chloride Trisodium citrate was administered at a starting rate of 4.3 mmol/L of extracorporeal blood flow. Citrate infusion rate was then adjusted to maintain the serum ionized calcium concentration < 0.3 mmol/L in the circuit. The CaCl_2_ replacement solution (1 g/10 mL) was administered via a central line at an initial rate adjusted to the citrate rate. The CaCl_2_ infusion rate was then adjusted to maintain the patient ionized Ca concentration in the normal range (1.05–1.15 mmol/L).

### 2.3 Calculations

From these data, base deficit, anion gap (AG), apparent and effective Strong Ion Difference (SIDa, SIDe), and strong ion gap (SIG) were calculated as described previously.^[6]^ In brief, 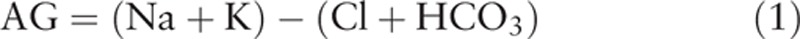 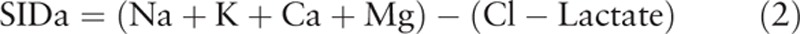 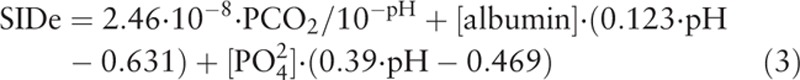 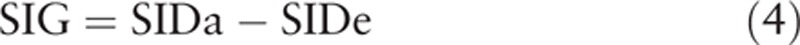


### 2.4 Statistical analysis

Patients were divided into 2 groups: survivors and nonsurvivors. The temporal evolution (Day 3–Day 1) of acid–base variables was analyzed in the 2 groups. In-hospital survivorship was assessed for up to 28 days after the start of CVVHDF. All statistical analyses were performed using a statistical software package (SPSS 10.0.5; SPSS Inc, Chicago, IL). Values are given as mean ± SD or median with interquartile range (IQR). Normal distribution of continuous variables was verified (Klomogorov–Smirnov test). Difference testing between groups was performed using the 2-tailed *t* test, χ^2^ test and Fisher exact test, Mann–Whitney *U* test as appropriated.^[10]^ Finally, receiver operator characteristics (ROC) curves were constructed to analyze each variable's discriminating power for predicting mortality and the areas under each curve were compared.

## 3 Results

Over the period analyzed, 200 subjects qualified for inclusion. We analyzed data from 245 subjects, 45 were excluded; 23 subjects did not have all the laboratory data required for acid–base quantitative analysis and in 22 the CVVHDF last <72 hours.

Demographic and acid base variables are shown in Table 1. In the first day of CVVHDF, subjects exhibited moderate acidemia (pH = 7.31 ± 0.11), and this was primarily due to hyperchloremia. There was no difference in the main demographic and acid base variables between those who died and those who survived to hospital discharge, except for a small increase in serum lactate levels in the nonsurvivor group (Table 1).

**Table 1:**
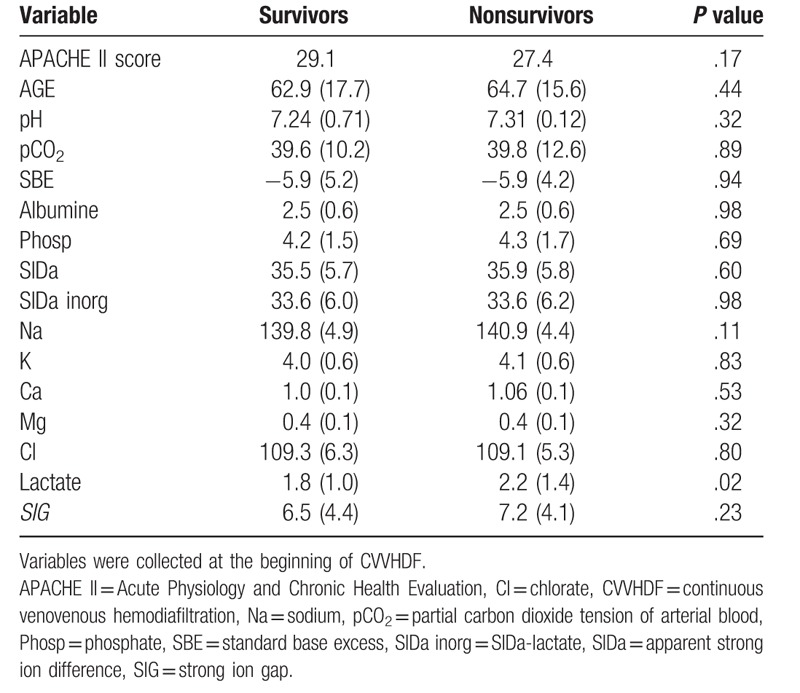
Demographic and acid–base characteristics in the first day of CVVHDF.

**Figure 1:**
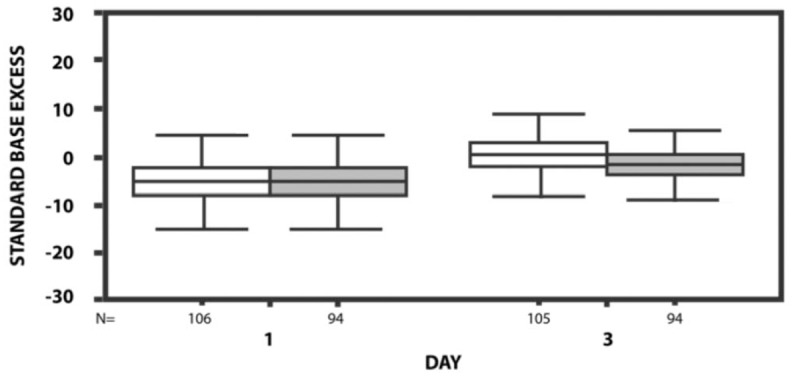
Temporal standard base excess evolution between survivors (white) and nonsurvivors.

However, by day 3 of CVVHDF there was a significant difference between the 2 groups (survivors and nonsurvivors) with respect to acid–base status (Table 2). Indeed, by the end of 72 hours the nonsurvivor group still exhibited metabolic acidosis (standard base excess (SBE): −2.7 +/− 4.0) while metabolic acidosis had resolved in the survivor group (SBE: −0.4 +/− 4.2) (Fig. 1). ROC curves for SBE proportional variation, lactate and SBE at day 3 were significant (*P* < .05) and are shown in Table 3.

**Table 2:**
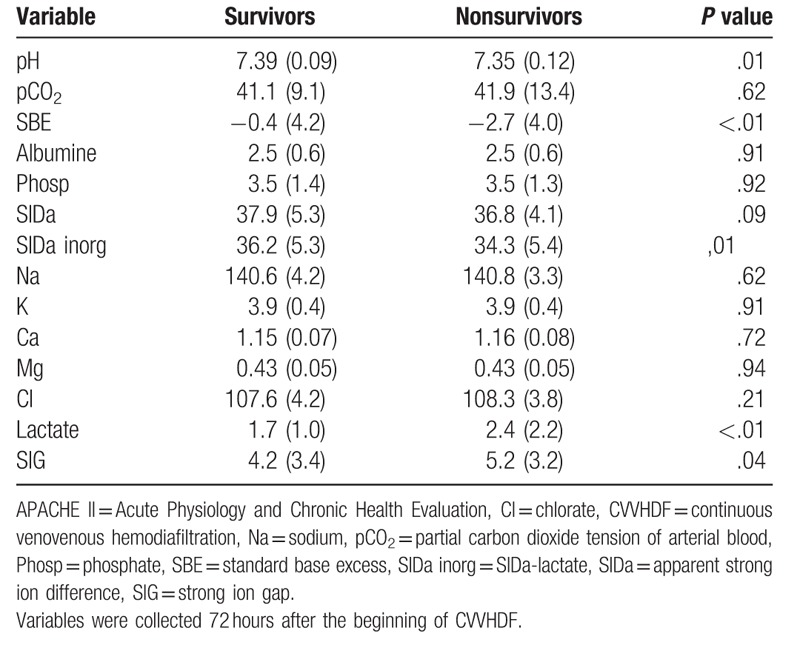
Acid–base characteristics in the third day of CVVHDF.

**Table 3:**
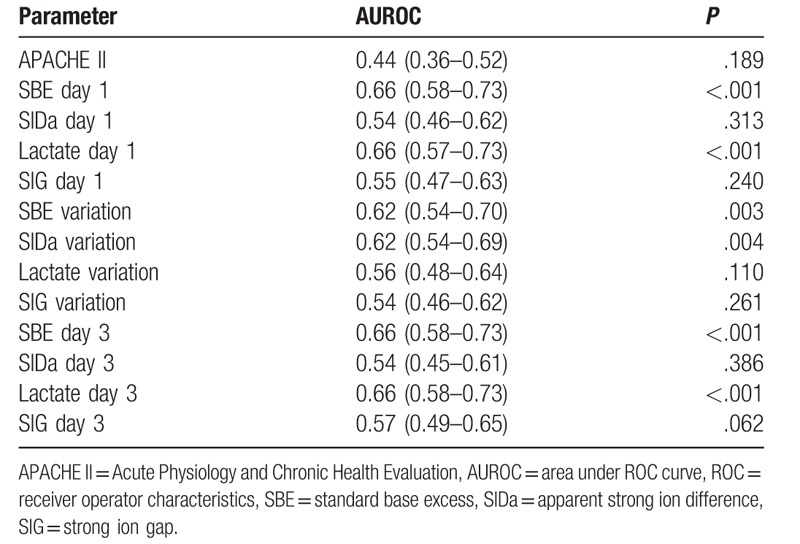
Summary of the ROC curves.

The main determinants of the change in acid–base variables over days 1–3 between survivors and nonsurvivors are shown in Table 2. Applying quantitative approach it was possible to determine which were the determinants of SBE evolution and consequently, which were the components responsible for the different patterns observed. Both survivors and nonsurvivors had similar resolution of SIG (−2.2 vs −2.0; *P* = .643) and neither exhibited much change in lactate (−0.1 vs 0.2; *P = *.127). By contrast chloride and sodium levels were the main determinants of the difference in SBE evolution between groups, accounting for 44% and 31% of this difference. However, neither of these reached statistic significance when considered alone (*P = *.267 and *P = *.174, respectively). However, the change in SIDa was significantly different between survivors and nonsurvivors (+2.5 vs +0.5; *P = *.01) (Table 4).

**Table 4:**
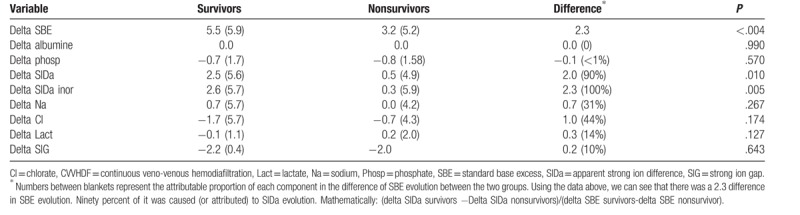
Metabolic acid base evolution during CVVHDF.

## 4 Discussion

Continuous renal replacement therapies, particularly CVVHDF, have been increasingly used in the treatment of critically ill patients with AKI. One important goal of renal replacement therapy is to correct acid–base disturbances, especially to avoid the possible detrimental effects of acidemia.^[1,2]^ However this effect is not achieved in all patients uniformly.^[4]^ In our study, nonsurvivors remained significantly acidotic after 72 hours of renal replacement therapy. By contrast survivors resolved their acidosis, mainly due to a change in SIDa, specifically caused by changes in chloride and sodium. These results are in accordance with other studies using different renal replacement therapies and different populations.^[11,12]^

However, the mechanisms behind these changes in acidosis over time are unclear. It does not appear that renal replacement therapy itself is responsible for the persisting hyperchloremic acidosis since all patients received renal replacement with the same composition of solutions and the doses of hemodiafiltration were similar in both groups. Furthermore, since the sieving coefficient of chloride is >1, chloride can be easily removed with renal replacement therapy.^[13]^

Hypercloremic acidosis in critically ill patients is multifactorial, but due in part to hyperchloremic resuscitation fluids like 0.9% “normal” saline (NS).^[14–17]^ Also endogenous hyperchloremia, attributable to Cl-shifts from extravascular to vascular spaces may also be seen, particularly in patients with sepsis.^[18,19]^ In our cohort, nonsurvivors had a significantly higher level of lactate, so more NS fluid administration might have been used to reverse presumed septic shock and lactic acidosis at the expense of hyperchloremia. We are unable to test this hypothesis within the restrictions of our database.

The finding that hyperchloremic acidosis was associated with worse outcome is also notable because this form of acidosis is not clearly as hazardous from a clinical perspective^[20]^ although animal studies have shown deleterious effect of hyperchloremia in experimental sepsis^[21,22]^ and in agreement of this recent studies in human reported similar findings.^[18,23–25]^

We found the SBE and lactate variation resulted in similar mortality prediction capability with area under ROC curve (AUROC) under the conventional threshold of 0.7 that is typically considered satisfactory for clinical use. These findings were consistent with the BEST Kidney study that tested the discrimination and calibration of several AKI scoring systems in a broad cohort of patients from multiple countries. In that study the lactate level achieve an AUROC of 0.639, which was similar to the more complex scoring systems.^[26]^ However, we consider that inadequate temporal evolution of SBE and lactate during CVVHDF should prompt the clinician to initiate both diagnostic and therapeutic actions. Page et al^[5]^ have shown in a retrospective study in a population of refractory septic shock that lack of metabolic improvement after 12 hours of CVVHDF, that was defined as unchanged base deficit despite the buffering action of CVVHDF, was associated with a 100% mortality rate. Furthermore, Passos et al^[27]^ have found an association between lactate clearance and lactate at 24 hours with mortality in patients with septic AKI undergoing CVVHDF. However, unlike in our study, the most important component of acidosis in theirs population was lactate.

SIG has recently been shown to predict outcome in various populations, particularly when measured early in the course of illness.^[28–31]^ One possible explanation for the poor discriminative ability of the SIG in this study is that correction of acidemia over CVVHDF is associated with a decreased SIG, in our study the temporal decrease of SIG was in parallel between survivors and non survivors. Rocktäschel and coworkers^[32]^ studied the effect of CVVH on acid–base balance. CVVH appears to correct metabolic acidosis in AKI through its effects on unmeasured anions, phosphate, and chloride. Passos et al^[33]^ did a similar study during CVVHDF with regional citrate anticoagulation and have found similar results. In both studies hypoalbuminemia had an alkalinizing effect.^[32,33]^

Our study has several limitations. First, as a retrospective study, our database is limited to the variables that were collected for clinical management. However, our inclusion criteria were predefined and data were retrieved from an independent computerized database. We only analyzed the first and third day of CVVHDF. Acute Physiology and Chronic Health Evaluation (APACHE) were recorded only on admission to ICU. We did not have data about differences in residual diuresis and the saline volume of resuscitation in the 2 groups.

## 5 Conclusion

The use of quantitative acid–base chemistry can discriminate the components of acidosis in patients with acute renal failure during CVVHDF. Survivors recover hyperchloremic metabolic acidosis more rapidly than nonsurvivors. Temporal evolution of SBE and lactate can still be used as monitoring tools in the population of ARF patients during CVVHDF. Further studies are needed to determine if survival can be improved by therapeutic measures aimed to correcting acidosis.

## Acknowledgments

We would like to thank Francisca Pereira Almeida that helped us to collect some data from our electronic ICU database.

## Author contributions

Rogério da Hora Passos (1) Design of study, collect data, Analysis of data and write the article.

Juliana Ribeiro Caldas Analysis of data and write the article.

João Gabriel Rosa Ramos Analysis of data and write the article.

Paulo Benigno Pena Batista Analysis of data and write the article.

Danilo T Noritomi Analysis of data and review the article.

Nelson Akamine Analysis of data

Marcelino S Durão Nephrology team, review the article.

Bento Fortunato Cardoso dos Santos Nephrology team, review the article.

Virgilio Gonçalves Pereira Junior Nephrology team, review the article.

Julio Cesar Martins Monte Nephrology team, review the article.

Marcelo C Batista Nephrology team, analysis of data and review the article.

Oscar F. P. dos Santos Nephrology team, review the article.

**Conceptualization:** Rogerio Passos.

**Formal analysis:** Rogerio Passos, Juliana Caldas, Joao Gabriel Rosa Ramos, Paulo Benigno Pena Batista, Danilo Teixeira Noritomi, Nelson Akamine.

**Investigation:** Rogerio Passos, Marcelino S Durao, Bento FC Santos, Julio CM Monte, Marcelo C Batista, Oscar FP Santos.

**Methodology:** Rogerio Passos.

**Project administration:** Rogerio Passos.

**Supervision:** Rogerio Passos.

**Validation:** Rogerio Passos, Juliana Caldas.

**Writing – original draft:** Rogerio Passos, Juliana Caldas, Joao Gabriel Rosa Ramos, Paulo Benigno Pena Batista, Marcelino S Durao, Virgilio G P Junior, Marcelo C Batista, Oscar FP Santos.

**Writing – review & editing:** Rogerio Passos, Joao Gabriel Rosa Ramos, Virgilio G P Junior, Marcelo C Batista.
